# Challenges of osteosarcoma care in Africa: a scoping review of the burden, management and outcome

**DOI:** 10.3332/ecancer.2025.1835

**Published:** 2025-01-23

**Authors:** Oluwaseun J Olajugba, Emmanuel O Oladeji, Damilola Adesola, Ridwanullah O Abdullateef, Godwin Rockson, Abdul K Bah, Oluwatobi O Olayode

**Affiliations:** Trauma and orthopaedics, Surgery Interest Group of Africa, Lagos 105101, Nigeria

**Keywords:** osteosarcoma, oncology care inequity, Africa, SDG, public health

## Abstract

Osteosarcoma has the highest incidence among individuals of African descent, with growing evidence suggesting ethnic and racial genetic underpinning. Hence, it presents a grave public health challenge in Africa given the widening inequities in access to cancer care. This scoping review addresses the critical gap in the availability of locally relevant data on the magnitude of the burden and challenges relating to the management and outcome of African centres. This study included 1,374 patients from eighteen studies. 81% presented with locally advanced or metastatic disease. While surgical treatment for osteosarcoma is shifting toward limb salvage on a global scale, amputation remains preponderant in Africa as only 53% underwent limb salvage operations. The pooled 5-year overall survival was 49.1%. Late presentation, workforce and infrastructural shortage, cultural beliefs, patronage of unorthodox medicine practitioners and high healthcare costs were the barriers driving poor outcomes in African centres. Strategies to improve outcomes should focus on addressing these barriers.

## Background

Globally, osteosarcoma is the most prevalent form of primary bone malignancy in children and adolescents, with an annual incidence of 5.6 cases per million children and a peak incidence in the second decade of life [1, 2]. It predominantly affects the extremities, threatening life and limb [2]. 

Although rare on a global scale, the incidence is highest among individuals of African descent, with growing evidence suggesting ethnic and racial genetic underpinning [3, 4]. Hence, it poses a grave public health challenge in Africa as the inequities in access to cancer care continue to widen [1, 2, 5, 6]. 

The clinical presentations of osteosarcoma are heterogeneous, and so are the imaging, pathological and biological behaviours [2]. Advances in cancer chemotherapy and surgical oncological techniques over the past decades have transformed the care delivered to osteosarcoma patients with a global trend toward limb salvage surgeries and overall improved survival outcomes and quality of life for patients [2]. The experience is however different in Africa where presentation with advanced disease remains rife, mortality is high and the limb is often beyond salvage [7]. 

Despite the increasing health burden, osteosarcoma, such as other cancers, receives distressingly little attention from continental and global policymakers and easily gets overshadowed by the health burden of infectious diseases [1, 8]. This is partly reinforced by the limited availability of locally relevant data on the magnitude of the problem and its impact on victims and their families. This lack of information tends to obscure the severity of the problem, underscoring the limited attention paid to addressing this burdensome socio-economic and public health problem, and the inherent tendency to undermine Goal 3 of the Sustainable Development Goals (SDG) [9]. 

To address this critical gap and identify the unique challenges that hinder the delivery of quality care to patients with osteosarcoma in African centres, we conducted a scoping review evaluating the burden of osteosarcoma in Africa, the management practices and the management outcomes. In addition to closing identified gaps in the literature, this review hopes to impact local policy and practice in African centres by providing further insights to guide healthcare resource allocation, evidence-based decision-making and policy impact assessment. We present this article following the preferred reporting systems for systematic review and meta-analysis extension for scoping reviews (PRISMA) guidelines.

## Methods

An electronic database search was performed on PubMed, African Journal Online, Web of Science, Google Scholar, Cochrane Library and Scopus using a combination of entry terms, text words and MeSH terms about the burden, management and outcome of osteosarcoma in African centres, from database’s inception to 30 June 2023. A manual search of the reference lists of identified articles was conducted to obtain relevant additional literature. 

Published articles retrievable in English on the burden, management and outcome of osteosarcoma of the appendicular skeleton were included in the review. Articles not available in full text, comments, letters to the editor, opinion pieces, case reports and reviews were excluded. Titles and/or abstracts of all relevant studies returned from the different databases were deduplicated and screened independently by two reviewers, followed by full article screening. A third reviewer resolved any disagreements between the first two reviewers. 

Relevant data were extracted in two stages – a pilot stage and the main stage, using a data extraction proforma developed on Microsoft Excel. Pooled statistics related to different characteristics were estimated. The review adopted the methodological guidance proposed by Peters *et al* [10] in the scoping review process. Neither ethical approval nor informed consent was required for this study.

## Results

**Figure 1.**
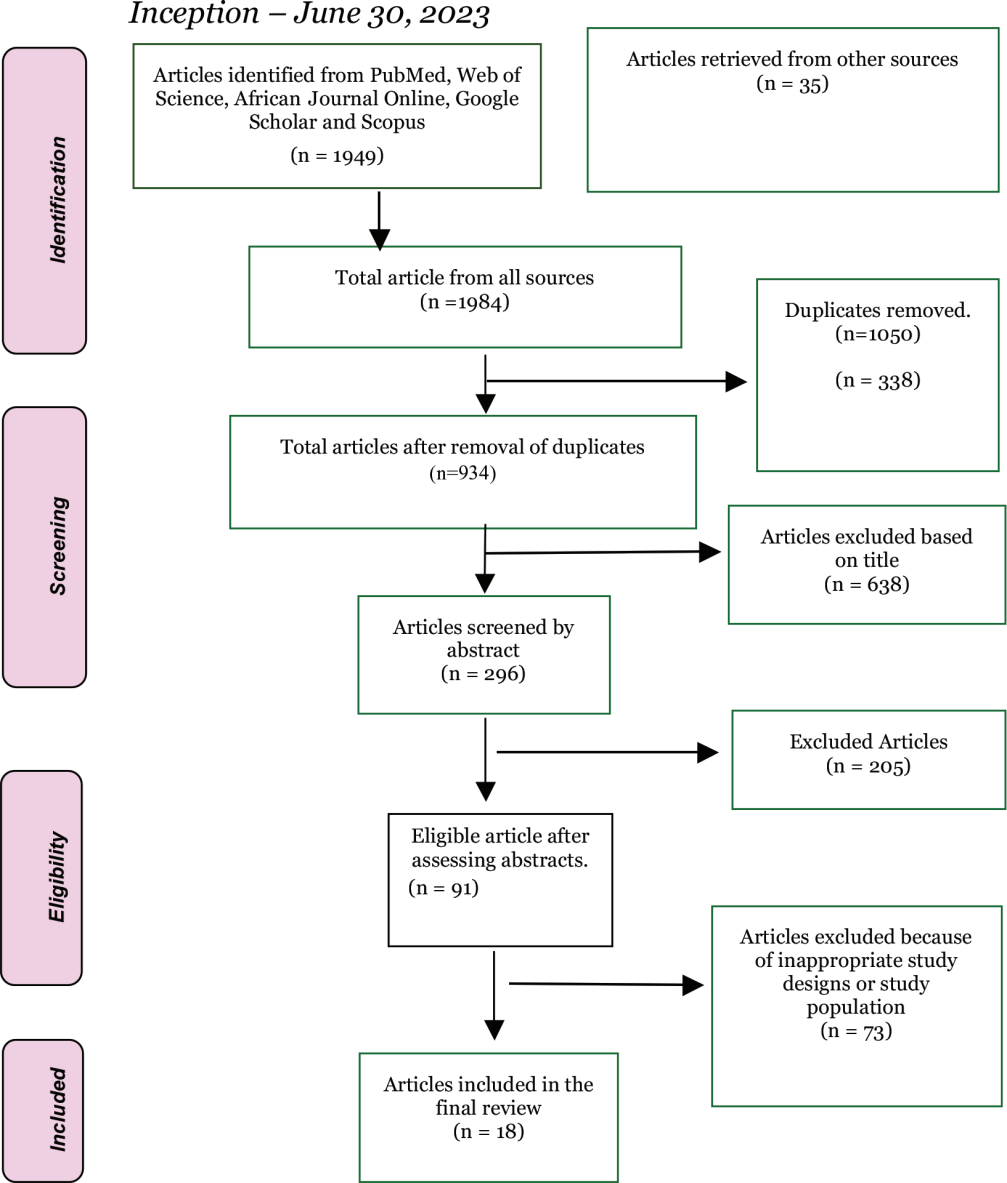
PRISMA flowchart of article identification and selection process.

Eighteen papers were eligible for inclusion in this review. A summary of the sequential screening process for article selection is presented in Figure 1. 

**Figure 2.**
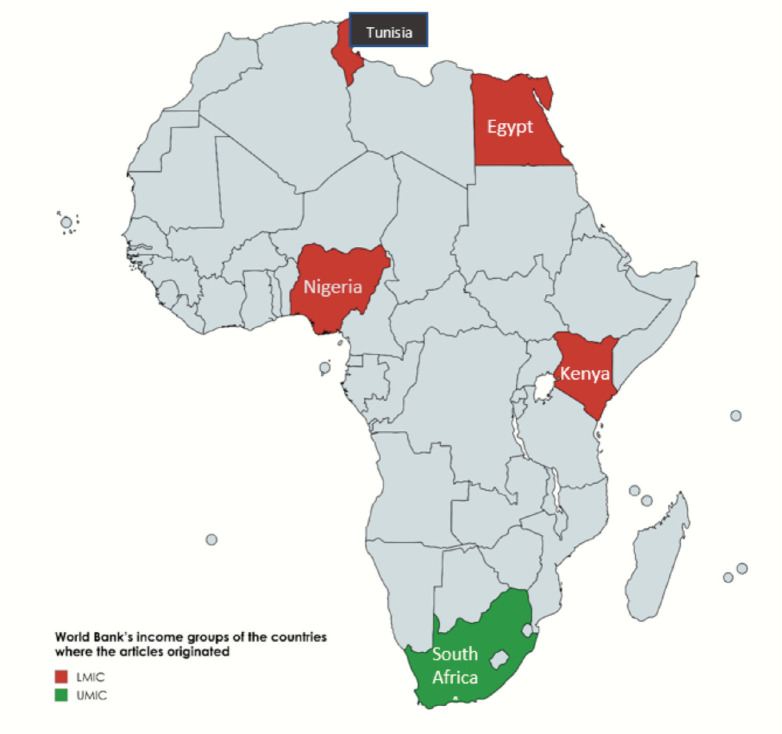
Map showing countries where the articles in this review originated and their income classification. LMIC: Lower middle-income country, UMIC: Upper middle-income country.

The majority of the papers were from Egypt (*n* = 9) and South Africa (*n* = 4), while Tunisia (*n* = 3), Kenya (*n* = 1) and Nigeria (*n* = 1) contributed the remaining papers. (Figure 2) The articles were published over 35 years, from 1985 to 2020, with most of them (*n* = 15) being published after 2010 (Table 1). All the articles were observational studies and the majority (*n* = 15) were conducted retrospectively (Table 1). 

A total of 1,374 patients from the eighteen eligible studies were included in this review (Table 1). The incidence of osteosarcoma was reported in only three studies and ranged from 1.2 to 3.2 cases per million population [11–13] Twelve studies reported the mean age of the study participants ranging from 13 to 42 years [11, 13–23] and the pooled mean was 17.6 years. Fifteen studies reported the sex distribution of the study participants, showing a pooled male (*n* = 508) to female (*n* = 452) ratio of 1.1 (Table 1).

Thirteen papers reported the anatomical location of osteosarcoma among the study participants (*n* = 572). The femur was the most affected in 289 cases, the tibia came a distant second (*n* = 166), while the axial skeleton was involved in 41 (7.2%) persons (Table 2). Pain, swelling, constitutional symptoms, ulcers and pathological fractures were the reported clinical features at presentation [14, 16, 22, 23]. The mean time from onset of symptoms to presentation was reported in five studies and ranged from 3.4 to 14.1 months with a pooled mean of 4.5 months [14–16, 22–24].

Data on the Enneking Staging System was available from seven studies. This review grouped the reported stages into localised (I and II) and metastatic (III) disease. 44.7% of the cases (*n* = 317) had the metastatic disease while the rest had localised disease – with the tumour locally advanced in about one-third (36.4%). The most common site of metastasis was the lungs. Using the adapted WHO classification of malignant primary bone tumours, [25] thirteen studies reported high-grade osteosarcoma (*n* = 482, 70.6%) as the most common pathological type among the study participants. 

Radiological assessment with plain radiographs was undertaken in all patients, however computerised tomography scan or magnetic resonance imaging (MRI) was conducted in only two-thirds of the participants. Additionally, 16 persons required further assessment with a bone scan and angiography [26, 27].

Therapeutic modalities were chemotherapy, surgery, radiotherapy, cryotherapy and palliative care. Surgical procedures reported from 9 studies include limb salvage operations in 53% and amputation in the rest (Table 3) [14, 16, 17, 23, 24, 27–29]. The details of limb salvage surgery undertaken were only available in three papers. This included wide excision with tumour prosthesis (with the use of megaprosthesis total knee replacement described in one), and tumour resection followed by reimplantation of frozen autograft [15, 23, 27]. Three patients with lung metastasis underwent pulmonary metastatectomy [16].

The chemotherapeutic regimens varied across studies with high-dose methotrexate (HDMTX) adopted in only one study (Table 3). None of the articles reported the use of a second-line chemotherapy regimen and details of radiotherapy treatment were lacking from papers that reported using this treatment modality. 

None of the papers suggested the involvement of multidisciplinary team discussion before the commencement of care. Eight studies reported refusal of treatment by the patient, carer or family representative in 14.6% of participants. The reasons for refusal of treatment included cultural beliefs, objecting to amputation, perceived futility of treatment and inability to afford the cost of care. 

Eleven of the eighteen studies reported survival outcomes. Outcome endpoints varied across studies and included overall survival (OS), event-free survival (EFS), disease-free survival (DFS) and progression-free survival (PFS) (Table 2). 3- and 5-year OS ranged from 40.7% to 79% and 38% to 78%, respectively, with a pooled OS of 49.1%, while the corresponding 3-year and 5-year EFS ranged from 43.5% to 70.5% and 40.7% to 65.2% [17, 28, 29]. Postoperative functional scores of 70%–82.4% were reported from two studies using a modified system of the Musculoskeletal System Society [26, 27]. 

Evidence of metastasis was the most common poor clinical prognostic factor, while high serum alkaline phosphatase and lactate dehydrogenase levels were identified as poor biochemical prognostic factors [14, 24, 29]. Additionally, a cytoplasmic pattern of Ephrin A4, high ADAM8 expression, down-regulated HLA Class I antigen expression and overexpression or co-expression of Ezrin and HER2 were the poor immunohistochemical prognostic factors [18–20, 26].

The timely management of patients who presented with osteosarcoma was hindered by several factors, including late presentation, poor access to oncology care, workforce and infrastructural shortage, cultural perceptions about limb loss leading to refusal of amputation, high patronage of unorthodox medical practitioners and spiritual healing centres, inability to afford the cost of care due to high out-of-pocket expenditure on healthcare and loss to follow up [14, 16, 17, 23, 24]. The mean interval from onset of symptoms to presentation was 4.5 months and limited access to MRI caused a delay in diagnosis and commencement of treatment [16]. Late presentation with advanced disease and pathological fracture was high and precluded limb salvage surgeries [14, 15]. Additionally, some patients had defaulted from treatment at a different hospital while others declined treatment for localised disease, only to present later with metastatic disease [14, 16].

## Discussion

This scoping review presents a summary of the current state of epidemiology, management practices and outcomes of osteosarcoma in Africa while highlighting the challenges that hinder the delivery of quality care. While previous reviews have addressed osteosarcoma burden and care from a global perspective, [2, 30] this is the first review to provide a summary of the literature from the African viewpoint. Despite the relatively higher disease burden of osteosarcoma in people of African descent [3], the vast majority of the studies identified during our literature search were conducted in high-income countries which reflects the generally low research output from Africa, especially in the sub-Saharan region [31]. This paucity of publications underscores the need to generate locally applicable and globally relevant data that can drive improvement in management practices and lead to better outcome profiles of patients managed for osteosarcoma in African centres.

Africa is a diverse continent with countries at various stages of cancer transition. Although sub-Saharan Africa bears a higher health burden of osteosarcoma, [8] most of the studies included in this review were conducted in Northern Africa, which may reflect the better allocation of resources for healthcare and cancer research in this African sub-region [5]. The incidence of 1.2–3.2 cases per million population found in this review is far lower than the 6.8 cases per million persons reported among African Americans by the US Cancer Statistics Working Group and the estimated 4.2 cases per million per year in sub-Saharan Africa reported by Rojas *et al* [30, 32]. This wide disparity mirrors the lack of effective population-based and hospital-based cancer registries in Africa in addition to the generally lower incidence of cancers in Northern Africa [5, 8, 33 34]. Although there were more males than females affected in our review, a male-to-female ratio of 1.1 suggests almost equal sex distribution, contrary to previous findings of male preponderance [2, 30, 35]. Deliberate investment in cancer registry and clinical services is key to obtaining accurate data on the magnitude of the disease and improving access to care. HDMTX chemotherapy regimen is an effective approach for treating osteosarcoma and is well-established in developed countries [36] Nevertheless, HDMTX therapy has a narrow therapeutic index and thus can cause significant toxicity leading to morbidities and occasionally mortality if MTX levels are not monitored rigorously [36]. This potentially deters using HDMTX in low-resource areas such as sub-Saharan African (SSA) and India, [37] as seen in this review where only two studies reported the use of MTX [24, 29], and just one of them adopted HDMTX (Table 3) [29]. Abdel Rahman *et al* [27] and Letaief *et al* [24] from studies conducted in Egypt and Tunisia, acknowledged the lack of resources for monitoring MTX levels thereby precluding HDMTX as a chemotherapy option in managing patients with osteosarcoma.

Access to oncological services is limited in Africa, which results in delayed cancer diagnosis and treatment [38–40]. This is due to an inadequate specialised workforce and deficient pathological, radiological and nuclear imaging infrastructures required to arrive at a diagnosis and properly stage cancers [16, 41, 42]. This was corroborated by Lisenda *et al* [16] who reported delays in diagnosis and treatment due to limited access to MRI. There is an acute shortage of MRI and computed tomography (CT) scanners in low- and middle-income countries (LMICs) with less than one CT scanner per 1 million population and an even wider gap for MRI machines [43]. This lack of workforce and infrastructure makes it challenging to complete the diagnosis timely [41, 44]. Building capacity through regional, continental and international collaborations is a proven strategy for tackling this problem.

Late presentation of cancer patients is an important problem in Africa, with the interval between the onset of symptoms and first presentation as high as 400 days in this review [24]. This may explain the high proportion of patients who presented with metastatic disease and the high rate of amputation performed instead of limb salvage operation which is now the global standard [2, 35]. 

The high cost of cancer care constitutes a major barrier to timely diagnosis, particularly in many SSA countries where patients incur out-of-pocket expenses for both direct and indirect costs of treatment [43, 45]. With little or no financial protection mechanisms, this situation is dire for many who live below the poverty line and families are hesitant to spend scarce resources on a disease that is perceived to be incurable in the first instance [43, 45]. This is comparable to the challenges faced in India in managing osteosarcoma as reported by Mittal *et al* [37] SSA and other LMIC countries such as India have poor surgical infrastructures, a shortage of skilled surgeons and require out-of-pocket healthcare financing, which can be a determining factor in accessing care by indigent patients [37]. 

Other reasons for delayed diagnosis include poor coordination between healthcare sectors and continued patronage of traditional healers and complementary medicine practitioners [43]. Addressing these barriers requires multidisciplinary policy formulation and commitment to implementation on a systemic level to achieve the SDG goal of reducing mortality from non-communicable diseases by one-third [9]. 

Of the 52 patients who refused treatment, 24 were offered amputation, either as a form of curative treatment or as part of a palliative care plan to treat pain and improve quality of life. Refusal to accept treatment suggests that even when confronted with a life-threatening condition, amputation as a treatment option is not culturally acceptable in some parts of Africa [46, 47]. Poor limb prosthetic services and the lack of well-coordinated pre- and post-amputation psychosocial support may additionally be the underpinning reasons why patients decline amputation [48]. Early presentation of patients and availability of specialised workforce and infrastructure for appropriate oncological surgery would significantly change the narrative toward performing more limb salvage operations. Culturally sensitive discussions with patients about treatment options by healthcare professionals can improve the treatment uptake.

In our review, the 5-year survival rate for osteosarcoma ranged between 38% and 78% with a pooled OS of 49.1%. This survival outcome is lower than survival rates of 60.17%, >65% and 68% reported from global studies [2, 30, 42]. It is important to note that most studies that reported outcome survival were from Northern Africa, where the provision of cancer care and availability of research resources appear better than most SSA. The implication is that the actual survival rate in Africa may be more disconcerting [8]. The prognostic factors identified in this review are consistent with findings from global studies [30, 42]. 

There were some limitations to this study. Most of the studies identified were hospital-based studies, predominantly from Northern and Southern Africa, which may have underestimated the burden of the disease. Also, only studies published in English were included in this review, potentially leaving out relevant information published in other languages. Additionally, case reports, conference proceedings and reviews were excluded, implying that relevant data may have been missed. One major challenge with making outcome comparisons between studies was the varying endpoints adopted by different authors, which were either not defined or only done vaguely, highlighting the need for transparent reporting of the component events and endpoints.

## Conclusion

This review highlighted the challenges compromising the management and outcome of osteosarcoma in Africa. While surgical treatment for osteosarcoma is shifting toward limb salvage on a global scale, amputation remains common in Africa and is an important reason why patients decline treatment. Late presentation, poor access to oncology care, workforce and infrastructural shortage, refusal of amputation, high patronage of unorthodox medical practitioners and spiritual healing centres and inability to afford the cost of care were the barriers limiting the delivery of appropriate care and driving the significant poorer outcomes. Overcoming these barriers will require a multifaceted approach matching policy formulation with sustainable implementation; human capital development with infrastructural upscaling; and international collaborations with optimisation of local resources.

## Recommendations

Addressing the barriers to osteosarcoma care in Africa requires a comprehensive and multifaceted approach incorporating targeted interventions at institutional, national and continental levels. A critical step in this direction is an accurate estimation of the disease burden to provide contextually appropriate data that would inform research priorities, allocation of resources and planning for future needs. Establishing and reinforcing cancer registries across Africa and fostering a culture of collaboration and information-sharing among researchers would ensure valuable data on incidence, outcomes and treatment efficacy are generated. 

Improved capabilities to expand access to appropriate diagnostic infrastructure is vital to ensuring timely diagnosis while expanding regional specialised oncology facilities and addressing the shortage of specialised healthcare workers. Creating partnerships with international organisations and non-governmental organisations can help equipment procurement and support training programs.

Chemotherapy is a cornerstone of osteosarcoma treatment. Hence, there is an imperative to standardise chemotherapy protocols to minimise variability in treatment strategies and ensure consistent, high-quality care across the continent. The limitation posed by the lack of capacity to monitor MTX levels in many African centres can be circumvented by adopting non-HDMTX regimens such as OGS-12 which has demonstrated comparable outcomes with acceptable toxicity [49]. Future research collaborations should explore the adaptation and effectiveness of these protocols in the African context.

Additionally, there is a need for enhanced capacity building through training programs for orthopaedic oncologists and other allied specialised healthcare workers, with a particular focus on limb salvage interventions that are feasible in low-resource settings, in addition to improving access to and the quality of prosthetic and psychological support services pre and post amputation. This can be achieved through partnerships between specialised centres across Africa and international collaboration initiatives.

Without mitigating the high cost of cancer care which typifies the lack of healthcare financial safety mechanisms in SSA countries, delayed diagnoses will continue to impact treatment outcomes adversely. Strategies to alleviate the high out-of-pocket expenses faced by patients and their families include increasing access to health insurance at national levels especially outside the formal sectors, adopting a plurality of health insurance schemes with each targeting different groups and innovative health financing strategies for cancer care through a partnership with international cancer care vanguards, industry and non-governmental organisations [50, 51]. 

Finally, improved awareness through educational campaigns and culturally sensitive dialogues highlighting the dangers of delayed or improper treatment targeting patients, communities in rural and underserved areas and primary healthcare providers are crucial to earlier detection and changing cultural perceptions and stigma surrounding the uptake of treatment modalities like amputation. 

By implementing these recommendations, African countries can build a sustainable framework to improve osteosarcoma care and outcomes, advancing both immediate patient support and a broader healthcare system.

## Conflicts of interest

The authors declare that they have no known competing financial interests or personal relationships that could have appeared to influence the work reported in this paper.

## Funding

This research did not receive any specific grant funding agencies in the public, commercial or not-for-profit sectors.

## Author contributions

Oluwaseun Joshua Olajugba and Emmanuel Olusola Oladeji contributed equally to this work. 

Oluwaseun Joshua Olajugba: Conceptualised the work, carried out literature search and data analysis, drafted the manuscript, critically revised it and approved it for final submission. 

Emmanuel Olusola Oladeji: Conceptualised and supervised the work, carried out literature search and data analysis, drafted the manuscript, critically revised it and approved it for final submission. 

Damilola Adesola: Data collection and analysis, critically revised the manuscript and approved it for final submission. 

Ridwanullah Olamide Abdullateef: Data collection and analysis, critically revised the manuscript and approved it for final submission. 

Godwin Rockson: Data collection, analysis, critically revised the manuscript and approved it for final submission. 

Abdul Karim Bah: Data collection, analysis, critically revised the manuscript and approved it for final submission.

Oluwatobi Olayode: Data analysis, critically revised the manuscript and approved it for final submission.

## Figures and Tables

**Table 1.** Study characteristics.

Author (s)	Study type	Countr y	Incidence (per million per year)	Sample size	Sex	Age (years)
Bovill *et al* [11]	Retrospective	Kenya	1.7	251	F = 141 M = 110	NA
Stefan *et al* [12]	Retrospective	Tunisia	1.2	359	NA	NA
Omonisi *et al *[13]	Prospective	Nigeria	3.2	4	F = 0 M = 4	NA
Marais and Ferreira [14]	Retrospective	South Africa	NA	8	NA	42
Ferreira and Marais [15]	Retrospective	South Africa	NA	24	F = 10 M = 14	20.8
Lisenda *et al* [16]	Retrospective	South Africa	NA	61	F = 20 M = 41	19.4
Morsy *et al *[17]	Retrospective	Egypt	NA	48	F = 27 M = 21	NA
Abdou *et al *[18]	Retrospective	Egypt	NA	47	NA	NA
Abd El- rehim and Osman [19]	Retrospective	Egypt	NA	61	F = 23 M = 38	24.6
Nada *et al* [20]	Retrospective	Egypt	NA	36	F = 9 M = 27	15.4
Abdou *et al *[21]	Retrospective	Egypt	NA	57	F = 19 M = 38	22
Ksontini *et al *[22]	Retrospective	Tunisia	NA	200	F = 110 M = 90	18
Shipley and Baukes [23]	Retrospective	South Africa	NA	30	F =11 M = 19	19.4
Letaief *et al *[24]	Retrospective	Tunisia	NA	85	F = 32 M = 53	17
Shalaby *et al *[26]	Case series	Egypt	NA	6	F = 3 M = 3	18.8
Abdel Rahman *et al *[27]	Prospective	Egypt	NA	10	F = 4 M = 6	21.1
Morsy *et al *[28]	Retrospective	Egypt	NA	30	F = 17 M = 13	NA
Zamzam *et al* [29]	Retrospective	Egypt	NA	57	F = 26 M = 31	13

F = female, M = male, NA = not applicable or not available

**Table 2.** Chemotherapy regimens and surgical procedures.

Authors	Number of patients in the study	Chemotherapy regimen	Type of surgery and number of patients
Marais and Ferreira [14]	8	Not reported	Amputation 6 (75%)
Ferreira and Marais [15]	24	Not reported	Amputation 6 (25%) Limb salvage 2 (8.3%): Wide resection and megaprosthesis TKR
Lisenda *et al* [16]	61	Not reported	Limb salvage 13 (21.3%) Amputations 33 (54.1%)
Morsy *et al* [17]	48	Doxorubicin (A)/Cisplatin (C) 3 preoperative cycles of A and C: A 75 mg/m^2^ and C 90 mg/m^2^ with a 3-week interval between cycles Same regimen postoperatively	Limb salvage 17 (35.4%) Amputation 13 (27.1)
Shipley and Beukes [23]	30	Not reported	Amputation 21 (70%) Limb salvage 2 (6.7%): Wide resection Excision + tumour prosthesis
Letaief *et al* [24]	85	Preoperative: Rosen T8–T12 regimensɫ OR API/AI regimens^ɸ^ OR Etoposide/Ifosfamide/Methotrexate regimen Regimen based on age: <18 years: Methotrexate-based regimens 18-25 years: Methotrexate-based regimens for the majority >25 years: API/AI regimen. Postoperative: Adjuvant chemotherapy depended on the tumour necrosis rate assessed by Huvos grading system	Limb salvage 34 (40%) Amputation 31 (36.5%)
Abdel Rahman *et al* [27]	10	Doxorubicin (A)/Cisplatin (C) 3 preoperative cycles of A and C: A 75 mg/m^2 ^and Cisplatin 150 mg/m2 Postoperative: Not reported	Tumour resection and reimplantation of frozen autograft 10 (100%)
Morsy *et al* [28]	30	Doxorubicin (A)/Cisplatin (C) 3 preoperative cycles of A and C: A 75 mg/m^2^ and C 90 mg/m^2^ with a 3-week interval between cycles Same regimen postoperatively	Limb salvage 12 (40%) Amputation 4 (13.3%)
Zamzam *et al *[29]	57	Preoperative: Two cycles of cisplatin 60 mg/m^2^ and doxorubicin 37.5 mg/m^2^ at weeks 1 and 6, AND Four cycles of HDMTX 12 g/m2 at weeks 4, 5, 9 and 10 with leucovorin rescue (15 mg every 6 hours, for 11 cycles, guided by MTX serum level). Postoperative: Eight cycles of high-dose MTX, two cycles of cisplatin/doxorubicin and two cycles of doxorubicin.	Limb salvage 47 (83%) Amputation 7 (12%) Rotationplasty 3 (5%)

ɫRosen T8–T12 regimens: Including Methotrexate, Cisplatin, doxorubicin, Bleomycin, Dactinomycin, and Cyclophosphamide.

^ɸ^API/AI regimens: Including Cyclophosphamide, doxorubicin, and ifosfamide

**Table 3.** Outcomes of care.

Author(s)	Enneking stage (number of patients)	Survival outcome (OS, EFS, PFS and DFS)
Marais and Ferreira [14]	I–II: 8 III: 16	No survival data
Lisenda *et al* [16]	I–II: 45 III: 16	Overall 1 year OS – 62.7% Overall 5 years OS – 38.1% IA: 5 years OS – 100% IIB: 1 year OS – 69.2% 5 years OS – 47.9% III: 1 year OS – 37.5% 5 years OS – 3.75%
Morsy *et al* [17]	I–II:30 III: 18	OS 65.2% at 3 years 65.3% at 5 years EFS 69% at 3 years 79% at 5 years
Abdou *et al *[18]	I–II: 20 III: 27	I-II: OS – 58% III: OS – 13.19%
Abd El-Rehim and Osman [19]	IA: 6 IB: 7 IIA: 10 IIB: 26 III: 12	Median OS – 25 months IA – 60 months IB – 48 months IIA – 57 months IIB – 47.1 months III – 18.1 months
Nada *et al* [20]	I–II: 27 III: 9	Mean OS 24.6 ± 8.1 months
Abdou *et al* [21]	I–II: 25 III: 32	Mean OS 27.86 ± 22.86 months
Ksontini *et al* [22]	NA	5 years OS – 78%
Shipley and Beukes [23]	I–II: 16 III: 14	Mean OS I–II – 30.3 months III – 9 months
Letaief *et al* [24]	I–II: 85 (all high-grade)	3 years OS – 49% 3 years EFS – 37% 5 years OS – 38% 5 years PFS – 32%
Shalaby *et al* [26]	IIb: 6	No survival data.
Abdel Rahman *et al* [27]	IIB: 10	Mean DFS – 54 months
Zamzam *et al* [29]	I–II: 57 (all non-metastatic)	3 years OS – 77.8% 3 years EFS – 70.5

OS = Overall survival, PFS = Progression-free survival, EFS = Event-free survival, DFS = Disease-free survival, Localised disease: Enneking I – II, Enneking Metastasis: III
